# Expression and distribution of the class III ubiquitin-conjugating enzymes in the retina

**Published:** 2010-11-18

**Authors:** Saima Mirza, Kendra S. Plafker, Christopher Aston, Scott M. Plafker

**Affiliations:** 1 Department of Cell Biology, University of Oklahoma Health Sciences Center, Oklahoma City, OK; 2 General Clinical Research Center, University of Oklahoma Health Sciences Center, Oklahoma City, OK

## Abstract

**Purpose:**

Mounting evidence implicates chronic oxidative stress as a significant pathogenic factor in the development and progression of retinopathies, including age-related macular degeneration (AMD). The age-dependent toxic accumulation of oxidatively damaged proteins, lipids, and DNA in susceptible cells of the retina arises, at least in part, from a decreased capacity to eliminate these damaged biomolecules. The goal of this study was to determine the expression patterns and function of class III ubiquitin-conjugating enzymes (UbcM3, UBE2E2, and UbcM2) in the retina. These enzymes have been implicated in the ubiquitin-dependent degradation of oxidatively damaged and misfolded proteins.

**Methods:**

Complementary western blotting and immunohistochemistry was performed with specific antibodies to determine the retinal cell expression pattern of each enzyme. Additional analyses using antibodies raised against UbcM2 were performed to determine the relative levels of the enzyme in lysates derived from various mouse organs as compared to the retina. An established light-damage model of oxidative stress-induced retinal degeneration was used to determine alterations in the susceptibility of mice harboring a single intact allele of UbcM2. Ubiquitin charging and auto-ubiquitylation assays were done to assess the catalytic state of UbcM2 following photo-oxidative stress.

**Results:**

Expression of the class III ubiquitin-conjugating enzymes in the retina, from highest to lowest, is UbcM2>UbcM3>UBE2E2. In addition to being the most robustly expressed, UbcM2 is further distinguished by its expression in photoreceptors and retinal pigment epithelial cells. UbcM2 is expressed in most mouse tissues analyzed and is most abundant in the retina. Studies using a bright-light-damage model of acute oxidative stress in mice harboring a single disrupted allele of UbcM2 revealed that a 58% reduction in enzyme levels did not increase the susceptibility of photoreceptors to acute photo-oxidative toxicity. This result may be explained by the observation that UbcM2 retained an intact and functional active site following exposure to acute bright light.

**Conclusions:**

The class III ubiquitin-conjugating enzymes, and in particular UbcM2, are expressed in the retina and may function to counter the accumulation of oxidatively damaged and misfolded proteins. A 58% reduction in UbcM2 does not increase the susceptibility of photoreceptors to an acute photo-oxidative stress, suggesting the existence of compensating enzymes and/or that the remaining UbcM2 activity is sufficient to target oxidatively damaged proteins for destruction.

## Introduction

The retina is highly susceptible to oxidative stress and damage due to its robust oxygen consumption, exceptionally high content of polyunsaturated fatty acids, and exposure to bright light. Together, these factors create a chronic oxidative burden that can result in damage to retinal proteins, DNA, and lipids [1]. Elimination of these oxidatively damaged biomolecules is required to prevent the toxicity that can result from their accumulation [2]. The accumulation of these damaged biomolecules is a hallmark of numerous neurodegenerative disorders, including age-related macular degeneration (AMD) [3]. The ubiquitin (Ub) proteolytic system (UPS) plays an integral role in destroying misfolded and oxidatively damaged proteins [4,5], and multiple lines of evidence implicate a critical function for this system in countering oxidative stress in the retina and lens. Evidence in support of this comes from studies showing that inhibition of the UPS in the retina, by either pharmacological means or with mutant Ub, leads to the deleterious accumulation of oxidized proteins [6,7].

The central player of the UPS is Ub, a highly conserved 76-amino acid polypeptide that is post-translationally attached to target proteins. Protein ubiquitylation is performed by an enzyme cascade consisting of a Ub-activating enzyme (E1), a Ub-conjugating enzyme (E2), and a Ub protein ligase (E3) [8]. In humans, there are two different E1s, at least 38 E2s, and 600–1,000 E3s [9]. Substrate selection and specificity are conferred primarily through the pairing of particular E2–E3 combinations. Ub is conjugated to an internal lysine of a target protein, and in the case of polyubiquitylation, subsequent Ubs are attached sequentially to a lysine of the previously added Ub. The best-studied fate of polyubiquitylation is that the modified protein gets targeted to the 26S proteasome for degradation. However, particular configurations of polyUb chains can result in non-proteolytic outcomes for the target protein. In addition, substrates can be regulated in non-proteolytic ways by the addition of a single Ub, a process referred to as monoubiquitylation. Analogous to the removal of phosphorylation by protein phosphatases, balance in the UPS is achieved by a set of Ub C-terminal hydrolases/deubiquitylating isopeptidases that cleave Ub from substrates (all reviewed in [10]).

Analysis of the retina for the expression and distribution of UPS components has demonstrated the presence of numerous enzymes in select cell types. For example, four different Ub-conjugating enzymes (E2_14K_, E2_20K_, E2_25K_, and E2_35K_) have been identified in bovine rod outer segments [11]. PGP 9.5, a Ub C-terminal hydrolase, is only present in retinal ganglion and horizontal cells [12], whereas the Ub hydrolase UCH-L3 is enriched in photoreceptor inner segments [13]. The E3 ligases Nedd4 and Siah1 have recently been identified in retinal ganglion cells and Müller cells, respectively [14,15]. Kelch-like 7 (KLHL7), a substrate adaptor for the cullin 3-based E3 ligase, is expressed in rod photoreceptors, and the identification of three different missense mutations has linked this protein to hereditary retinitis pigmentosa [16].

It is likely that these various enzymes represent only a small subset of the UPS components present in the retina. We undertook the present study to analyze the expression and distribution of the class III E2s in the retina. The human class III E2s are called UBE2E1, UBE2E2, and UBE2E3. The mouse versions are referred to as UbcM3, UBE2E2, and UbcM2, respectively, and each is identical to its human counterpart [17] (see Table 1). These enzymes share a host of properties, including: 1) a steady-state nuclear distribution [18,19], 2) entering the nucleus via the importin-11 transport receptor [18,19], 3) binding common E3 ligase partners (e.g., [20,21]), and 4) interacting with the N-terminal domain of various cullin proteins [21]. An additional distinguishing feature among these enzymes is that each has a unique N-terminal domain of 40–60 residues [17]. The rationale for analyzing the retinal expression patterns of these enzymes is threefold. First, these enzymes are functional homologs of a pair of *S. cerevisiae* E2s, Ubc4, and Ubc5, that mediate the degradation of misfolded and oxidatively damaged proteins [17,22-24]. Second, we recently reported that these enzymes can directly bind the master antioxidant transcription factor Nrf2 [25]. Moreover, we demonstrated that UbcM2 can stabilize and transcriptionally activate Nrf2 and that these functions are largely mediated by a unique cysteine residue present in the class III E2s [25]. In the context of the retina, Nrf2 has been shown to play an important role in conferring protection from photo-oxidative and electrophilic stress [26-34]. Third, we have shown in cultured retinal pigment epithelial cells that UbcM2 is required for proliferation [35]. Depletion of the enzyme by small interfering RNA (siRNA) caused a robust increase in the cell-cycle inhibitor p27^Kip1^. However, the mechanism underlying this cell-cycle effect is unknown.

**Table 1:** Class II E2 nomenclature.

**Mouse**	**Human**
UbcM3	UBE2E1, UbcH6
UBE2E2	UBE2E2, UbcH8
UbcM2	UBE2E3, UbcH9, E2–23K, UBCE4

Using antibodies specific for each enzyme, we have discovered that these three enzymes display differential distributions in the mouse retina. Interestingly, only UbcM2 was detectable in the nuclei of retinal pigment epithelial cells and in photoreceptors. Furthermore, we demonstrate that UbcM2 is expressed in most mouse tissues analyzed but is most abundant in the retina. Studies using a bright-light damage model of acute oxidative stress in mice harboring a single disrupted allele of UbcM2 revealed that a 58% reduction in enzyme levels did not increase the susceptibility of photoreceptors to photo-oxidative toxicity. These light-damage studies further revealed that UbcM2 retains its catalytic capacity following exposure to acute bright light. Together, these studies are the first to describe the retinal expression pattern of the class III E2s and to test the concept that a full complement of one of these enzymes, UbcM2, is necessary to protect photoreceptors from an acute oxidative insult.

## Methods

### Antibodies

Anti -UbcM3, anti -UBE2E2, and anti -UbcM2 were raised in rabbits against recombinant His_6_-S-tagged polypeptides corresponding to the unique N-terminal extension of each enzyme. The His_6_-S tag consists of a hexa-histidine stretch followed by the S-peptide from RNase A, and the combination is encoded in the series of pET30 vectors from Novagen EMD Biosciences (Darmstadt, Germany). Purified glutathione S-transferase (GST) fusions of each enzyme were used for affinity purification. anti-glutathione S-transferase (anti-GST) was purchased from Bethyl Laboratories (Montgomery, TX) and anti-UbcH6 from Boston Biochem (Cambridge, MA).

### Western and dot blotting

GST- and His_6_-S-tagged recombinant fusion proteins were expressed from pGEX-2T and pET30a, respectively, and purified from BL21(star) *E. coli,* as described previously [21]. For the dot blot assays, five sheets of nitrocellulose were spotted with 10, 1, or 0.1 ng of either GST, GST-UbcM3, GST-UBE2E2, or GST-UbcM2 and then air dried. Blots were blocked for 60 min in 5% nonfat dried milk in TBST (20 mM Tris-HCl [pH 7.4], 150 mM NaCl, 0.1% Tween-20) and incubated with primary antibodies diluted as follows: anti-GST (1:1,000), anti-UbcM3 (1:100), anti-UBE2E2 (1:100), anti-UbcM2 (1:1,000), and anti-UbcH6 (1:1,000). Primary antibodies were detected with a horseradish peroxidase-conjugated goat antirabbit secondary antibody diluted at 1:2,500 followed by enhanced chemiluminescence.

### Small interfering RNA transfections

Synthetic pools of UbcM2-specific or UbcM3-specific siRNAs as well as control siCON siRNA (all from Dharmacon, Inc.) were combined with oligofectamine (Invitrogen, Carlsbad, CA) for 20 min before being added to 5×10^5^ HeLa cells in 12-well dishes. A final concentration of 60 nM siRNA was added to the cultured cells. Four hours following the addition of siRNA, 1 ml of DMEM (cat. # 10–013-CV; Mediatech, Inc.) supplemented with 10% fetal calf serum, 100 units/ml penicillin, and 0.1 mg/ml streptomycin sulfate was added to each well. Samples were harvested 72 h post transfection and processed for western blotting.

### Light-damage experiments

All animal care procedures complied with the ARVO Resolution on the Use of Animals in Research and with the rules and regulations of the OUHSC Institutional Animal Care and Use Committee. A modification of an established bright-light damage protocol was followed [36,37]. Six-week old, albino SvEv mice (wild type [WT] and UbcM2^+/−^) [35] (Of note, this albino strain is a mixed SvEv/Bl6 line) that were born and raised in dim cyclic light (5 lux, 12 h:12 h on–off cycle) were dark adapted overnight before being exposed to 3,000 lux of white, cool, diffuse fluorescent light for 6 h in a light box with reflective surfaces and a wire top. Each cage housed a single animal, and animals had unrestricted access to food and water. Following the light stress, animals were returned to the dim cyclic light for 1 week before being sacrificed by asphyxiation with carbon dioxide. Negative control animals were treated identically except they were not exposed to the bright light. For the auto-ubiquitylation studies, animals were exposed to 3,000 lux for 6 h and then immediately sacrificed and retinas harvested for lysates. For immunohistochemical studies, enucleated eyes were fixed overnight at room temperature in Perfix (20% isopropanol, 2% trichloroacetic acid, 4% paraformaldehyde, and 2% zinc chloride) followed by embedding in paraffin. Five-μm-thick sections were cut along the vertical meridian and stained with hematoxylin and eosin. Starting at the optic nerve head (ONH) and extending to the superior and inferior ora serrata, outer nuclei layer (ONL) counts were collected at 225-μm intervals. Spider graphs were compiled from pooled data with error bars representing standard deviations.

### Immunohistochemistry

**Figure 1:**
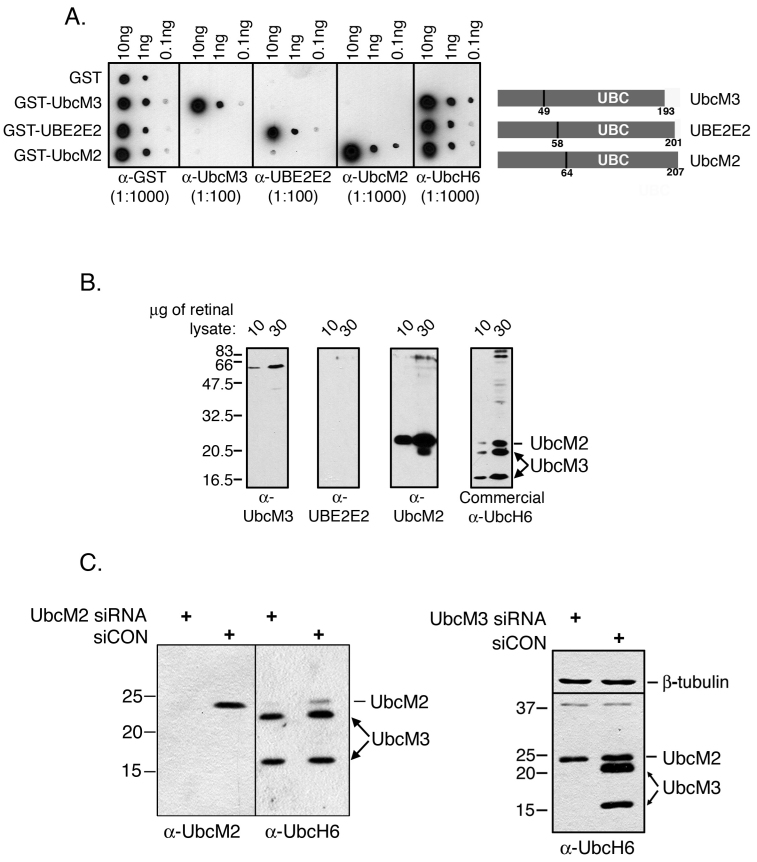
Characterization of the sensitivity and specificity of class III ubiquitin conjugating enzyme (E2) antibodies. **A**: Dot blot assay using recombinant glutathione S-transferase (GST) or GST-E2 fusion proteins. The indicated recombinant proteins (10, 1, or 0.1 ng) were spotted on pieces of nitrocellulose paper in quintuplicate. The blots were blocked in 5% milk/TBST and then incubated with anti-GST, anti-UbcM3, anti-UBE2E2, anti-UbcM2, or a commercial antibody against human UbcM3 (anti-UbcH6). To the right of the blots is a diagram of the class III E2s highlighting the relative location of the conserved catalytic core domain (UBC), the number of residues in each protein, and the residue corresponding to the end of the unique N-terminal extension. **B**: Mouse retinal lysate (10 or 30 μg) was resolved by sodium dodecyl sulfate PAGE (SDS–PAGE) in quadruplicate, transferred to nitrocellulose, and probed with the indicated antibodies. The migration of UbcM2 and UbcM3 is indicated to the right of the anti-UbcH6 blot. Two distinct isoforms of UbcM3 are detected (arrows). The migration of molecular weight markers is indicated on the left. **C**: siRNA experiments in HeLa cells to demonstrate that targeted knockdown of UbcM2 results in loss of the band denoted as UbcM2 but does not affect UbcM3 expression (left blot), and targeted knockdown of UbcM3 results in loss of detection of both isoforms of the enzyme (right blot). The migration of molecular weight markers is indicated to the left of the blots.

Heat-induced epitope retrieval (HIER) was performed on paraffin-embedded sections, using a pressurized Decloaking Chamber (Biocare Medical; Concord, CA) in citrate buffer (pH 6.0) at 99 °C for 18 min. Slides were incubated in 3% hydrogen peroxide, followed by normal serum and BSA at room temperature for 20 min each. After incubation with primary antibodies (anti-UbcM3 diluted 1:250; anti -UBE2E2 diluted 1:250; anti -UbcM2 diluted 1:2,000), the slides were incubated in polymer-horseradish peroxidase-conjugated secondary antibody (DAKO, Glostrup, Denmark) and developed with diaminobenzidine (Sigma, St. Louis, MO). Counterstaining was accomplished with analine blue (Sigma). Slides were examined with a Nikon 80i microscope and DXM1200C camera, and images captured using NIS-Elements software (Nikon; Tokyo, Japan). Image processing was done with Adobe Photoshop (version 8.0). Of note, the dilutions of antibodies used were based on pilot studies done to optimize the signal to noise ratio. Further, because anti-UbcM2 was more sensitive than anti-UbcM3 and anti-UBE2E2 (based on the GST-fusion dot blots [Figure 1A]), we diluted the anti-UbcM2 antibody 1:2,000 but the other two antibodies 1:250 to normalize for this difference.

### Mouse tissue analysis

The generation of SvEv/129 UbcM2 heterozygotes (UbcM2^+/−^) has been described previously [35]. Extracts were made by homogenizing tissues in Tissue Protein Extraction Reagent (ThermoScientific, Waltham, MA) followed by a 30-min incubation in a 4 °C thermomixer shaking at 1,000 rpms. Lysates were then clarified by centrifugation at 16,000× g in a 4 °C tabletop centrifuge. Protein concentrations of clarified lysates were determined by bicinchoninic acid assay (ThermoScientific). Retinas were isolated by the “Winkling” procedure [38]. Briefly, immediately following sacrifice, forceps were placed around the optic nerve and the eye was lifted slightly. A razor blade was used to cut the globe along the equator, thus permitting removal of the cornea and lens. Further raising of the eye with the forceps caused detachment and subsequent extrusion of the retina from the globe. Equal micrograms of retinal lysates were resolved by sodium dodecyl sulfate PAGE (SDS–PAGE) and processed for western blot analysis. Comparable loading of samples was monitored by amido black staining of the nitrocellulose membranes.

### Ubiquitin-charging and in vitro auto-ubiquitylation assays

#### Ub-charging assay

Mouse retinal lysates were isolated and protein concentrations determined as described above. Equal amounts of protein were then solubilized with nonreducing SDS–PAGE buffer (50 mM Tris-HCl [pH6.8], 4 M urea, 2% SDS, 10% glycerol, and 0.001% bromophenol blue) [39]. Subsequently, samples were heated for 15 min in a 30 °C water bath, and then one-half of each sample was transferred to a new tube and combined with 1 μl β-mercaptoethanol (14.3 M) to reduce the thiolester bond between the active site cysteine of UbcM2 and Ub. Thirty micrograms of each nonreduced and reduced sample in parallel was then resolved by SDS–PAGE in a 4 °C cold room at 100 V constant, electrotransferred to nitrocellulose at room temperature, and analyzed by anti-UbcM2 western blotting.

#### In vitro ubiquitylation assay

For each sample, 50 μg of clarified retinal lysate was combined with anti-UbcM2 and protein A Sepharose for 2 h at 4 °C. Immunoprecipitations were washed with 20 column volumes of ice-cold wash buffer (10 mM HEPES-KOH [pH 7.4], 55 mM potassium acetate, 1 mM magnesium acetate, 0.1 mM EGTA, 0.25% Tween-20, 150 mM NaCl) before being combined with a ubiquitylation cocktail containing recombinant human E1 (0.14 μg/reaction; Boston Biochem), Ub (16 μg/reaction), and an energy-regenerating system (100 mM Tris-HCl [pH 7.4], 0.4 mM MgATP (from 100 mM stock of MgATP using 100 mM magnesium acetate, 20 mM HEPES [pH 7.5], ATP and water. This stock was then diluted to a final concentration of 0.4 mM), 1 mM MgCl_2_, 0.2 mM DTT, 2mM phosphocreatine, 0.2% Tween-20, and 0.5 mg creatine phosphokinase) for 90 min at 37 °C. Reactions were terminated by the addition of concentrated (4×) Laemmli solubilizing buffer, resolved by SDS–PAGE, and analyzed by anti-UbcM2 western blotting.

### Statistical analyses for light-damage studies

Results were expressed as mean±standard deviation. The outcome of interest for the primary analysis was the amount of light damage measured for each animal in the light-damaged group as the ONL count at a particular position (relative distance from the optic nerve head) minus the average of the negative control animals of the same sex and genotype at that same position. The primary analysis determined whether there were significant differences in the amount of damage between the two genotypes: WT and UbcM2 heterozygotes (UbcM2^+/−^). Secondary analyses used the absolute ONL counts to determine sex and genotype differences between negative control animals. Analyses used generalized linear models with genotype and sex effects nested within position to allow for determination of significance of the overall sex or genotype effect, while accounting for differences between positions. These were followed by post hoc pair-wise tests within each site, with a Scheffe correction for multiple comparisons. Descriptive statistics were calculated in Microsoft Excel (Microsoft Corporation), while all statistical analyses used SAS (version 9.1, SAS Institute Inc., Cary, NC).

## Results

The human genome encodes at least 38 different E2s [9], but the tissue distributions, E3 ligase partners, substrates, and functions of many of these enzymes remain unknown. Of this large group of enzymes, the class III E2s (UBE2E1, UBE2E2, and UBE2E3) are of particular interest due to their high conservation among metazoans. For example, the respective mouse and human counterparts of all three enzymes are 100% identical [17]. In this study all of the work is done with the mouse enzymes, and we therefore refer to the enzymes and their respective antibodies by the mouse nomenclature (Table 1). UBE2E1 is referred to as UbcM3, UBE2E2 is UBE2E2, and UBE2E3 is UbcM2.

The high level of conservation (>95%) among the class III E2s suggests that these three enzymes have functional overlap and therefore might be distinguished by their respective tissue and/or cellular distributions. To address this, we developed rabbit polyclonal antibodies against the three enzymes, taking advantage of the fact that each possesses a unique N-terminal domain (Figure 1A). Antibodies were raised against recombinant His_6_-S fusions of each N-terminal domain and affinity purified using GST fusions of each. Dot blot assays using the GST-fusion proteins established the specificity and sensitivity of each antibody. Anti-UbcM3 and anti-UBE2E2 readily detected 1 ng of their respective GST fusions, whereas anti-UbcM2 detected as little as 0.1 ng of GST-UbcM2 (Figure 1A). None of the antibodies appreciably cross-reacted with GST fusions of the other two enzymes. An anti-GST control dot blot was done to show that equivalent amounts of the various fusion proteins were spotted. A commercial antibody, referred to hereafter as anti-UbcH6, raised against the human form of UbcM3 (also known as UbcH6) was also tested. This antibody detected all of the class III E2s, which precluded its use for subsequent immunohistochemical (IHC) studies. Of note, anti-UbcH6 has a higher sensitivity for GST-UbcM3 compared to anti-UbcM3.

As the retina is subjected to chronic oxidative stress [1] and the class III E2s are functional homologs of the yeast enzymes that degrade oxidatively damaged proteins [17,22-24], we next examined which of the enzymes is expressed in the mouse retina (Figure 1B). The three enzymes migrate at 20–25 kDa in SDS–PAGE. We found that UbcM2 was readily detectable but UBE2E2 was not. Using our anti-UbcM3 antibody, we did not detect UbcM3. In contrast the commercial anti-UbcH6 antibody detected UbcM3 as well as UbcM2 and a third faster migrating band that represented a second isoform of UbcM3. The identification of this faster migrating band as a second isoform of UbcM3 is based on siRNA experiments targeting the expression of either UbcM2 or UbcM3. Expression of the band identified as UbcM2 was reduced by UbcM2-specific siRNA (Figure 1C, left panel), whereas expression of the two UbcM3 bands was reduced by UbcM3-specific siRNA (Figure 1C, right panel). A control siRNA (siCON) did not affect the expression of either enzyme. The different results regarding UbcM3 expression in the retina using anti-UbcM3 and anti-UbcH6 are consistent with the higher sensitivity of anti-UbcH6 relative to anti-UbcM3 (Figure 1A). Further, the nearly comparable detection of both UbcM2 and UbcM3 by commercial anti-UbcH6 but the approximately tenfold higher sensitivity of the antibody for UbcM3 (Figure 1A) indicates that UbcM2 is expressed in the retina at higher levels than UbcM3. These data show that expression of the class III E2s in the retina, from highest to lowest, is UbcM2>UbcM3>UBE2E2.

**Figure 2:**
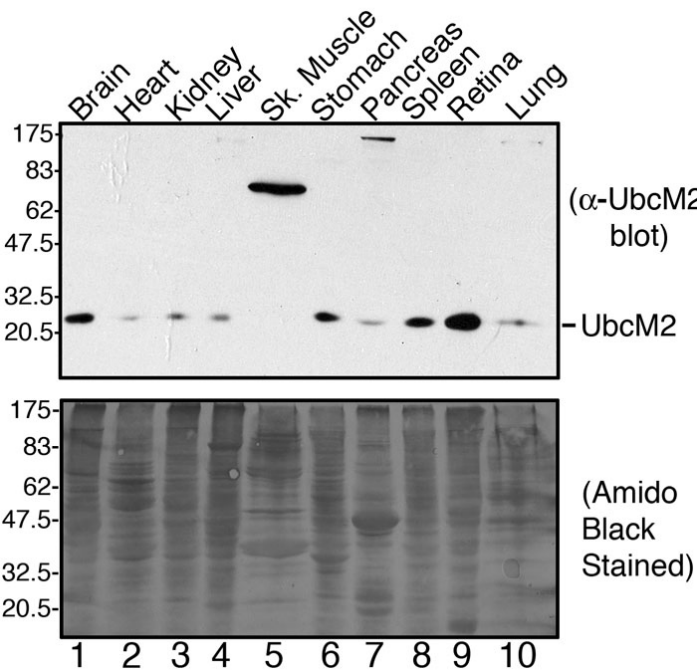
UbcM2 is enriched in the retina and expressed in most organs. Thirty micrograms of lysate from the brain, heart, kidney, liver, skeletal muscle, stomach, pancreas, spleen, retina, and lung of a 7-month-old mouse were subjected to anti-UbcM2 western blotting (top blot). The same blot was stained with Amido Black to demonstrate comparable protein levels in each lysate (bottom blot). The migration of molecular weight markers is shown to the left of the blots.

The high sensitivity of anti-UbcM2 prompted us to determine the tissue distribution of the enzyme and to compare expression levels to those detected in the retina. Various organs were harvested from a 7-month-old mouse, and 30 μg of each lysate was resolved by SDS–PAGE for western blotting with anti-UbcM2. Amido black staining of the blot was performed to demonstrate comparable loading. These experiments demonstrated that UbcM2 is most prominently expressed in the retina (Figure 2, lane 9). The enzyme is second-most prominently expressed in the brain and spleen (Figure 2, lanes 1 and 8, respectively), with lower amounts detectable in the heart, kidney, liver, stomach, pancreas, and lung (Figure 2, lanes 2, 3, 4, 6, 7, and 10, respectively). Strikingly, skeletal muscle lysates contained a prominent band migrating at ~75 kDa but had no band at the predicted migration for UbcM2 (Figure 2, lane 5). The identification of this 75-kDa band is unknown. These data reveal that UbcM2 is enriched in the retina and ubiquitously expressed at lower levels in most tissues and organs analyzed.

**Figure 3:**
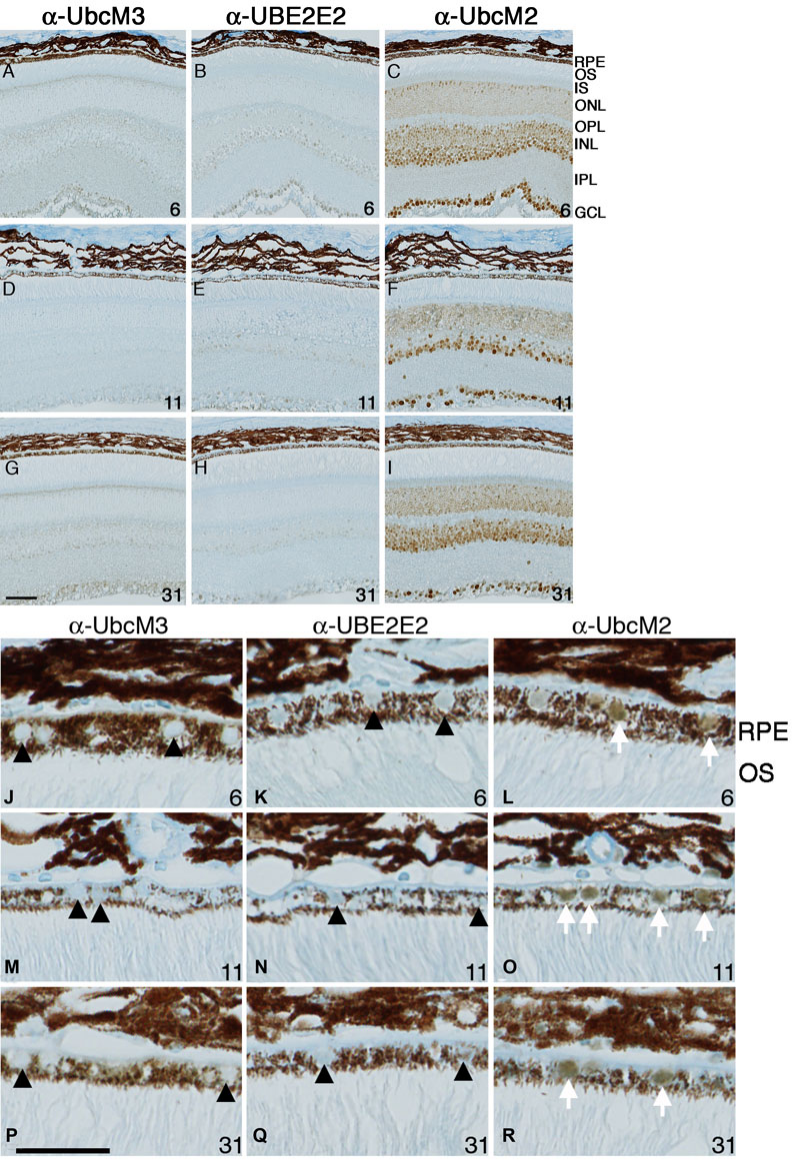
Differential expression of class III ubiquitin conjugating enzymes (E2s) in cells of the mouse retina. **A-I**: Shown here are representative photomicrographs of paraffin-embedded sections labeled with the indicated antibodies. Mouse age in weeks is shown in the bottom right corner of each panel. Abbreviations are as follows: RPE represents retinal pigment epithelium; OS represents outer segments; IS represents inner segments; ONL represents outer nuclear layer; OPL represents outer plexiform layer; INL represents inner nuclear layer; IPL represents inner plexiform layer; GCL represents ganglion cell layer. The magnification bar represents 50 μm, and all photomicrographs were taken at the same magnification. **J-R**: Enlarged sections are shown of images from (**J**) highlighting the presence or absence of RPE labeling with the various antibodies. Black arrowheads mark unlabeled RPE nuclei and white arrows indicate nuclei labeled with anti-UbcM2. The magnification bar represents 25 μm.

To corroborate and extend the findings from the retinal lysate western blots, we determined the temporal retinal cell expression pattern of UbcM3, UBE2E2, and UbcM2. To accomplish this, we performed IHC staining of paraffin-embedded sections of retinas from 6-, 11-, and 31-week-old mice. As we reported previously [19], all of the class III E2s have a steady-state nuclear distribution, and therefore we analyzed the sections for nuclear immunohistochemical staining. In addition we used anti-UbcM3 and anti-UBE2E2 at dilutions of 1:250 but anti-UbcM2 at a dilution of 1:2,000 as a means of normalizing the antibodies for their relative sensitivities (Figure 1A). These experiments demonstrated minimal nuclear staining for UbcM3 at all time points (Figure 3A,D,G), although we could detect faint cytoplasmic speckling in the inner segments of the photoreceptors and in the ganglion cell layer. Because UbcM3 is a nuclear enzyme [19], this labeling is likely to be nonspecific. Faint nuclear UBE2E2 was detected in some nuclei of the inner nuclear layer (i.e., second order neurons) and in ganglion cells (Figure 3B,E,H). A range of nuclear UbcM2 expression was detected in the inner nuclear layer, and robust staining was apparent in ganglion cells, whereas moderate UbcM2 expression was evident in the ONL (i.e., photoreceptor nuclei; Figure 3C,F,I). Closer examination of the retinal pigment epithelial (RPE) layer revealed nuclear staining for UbcM2 (Figure 3L,O,R), but not UbcM3 or UBE2E2, at all three time points (Figure 3J,K,M,N,P,Q). The relatively higher IHC detection of UbcM2 versus the other two enzymes is consistent with the western blotting results (Figure 1B). Together, these data show that, within the detection limits of the antibodies used, UbcM2 is enriched in the retina, especially in RPE cells, compared to UbcM3 and UBE2E2.

**Figure 4:**
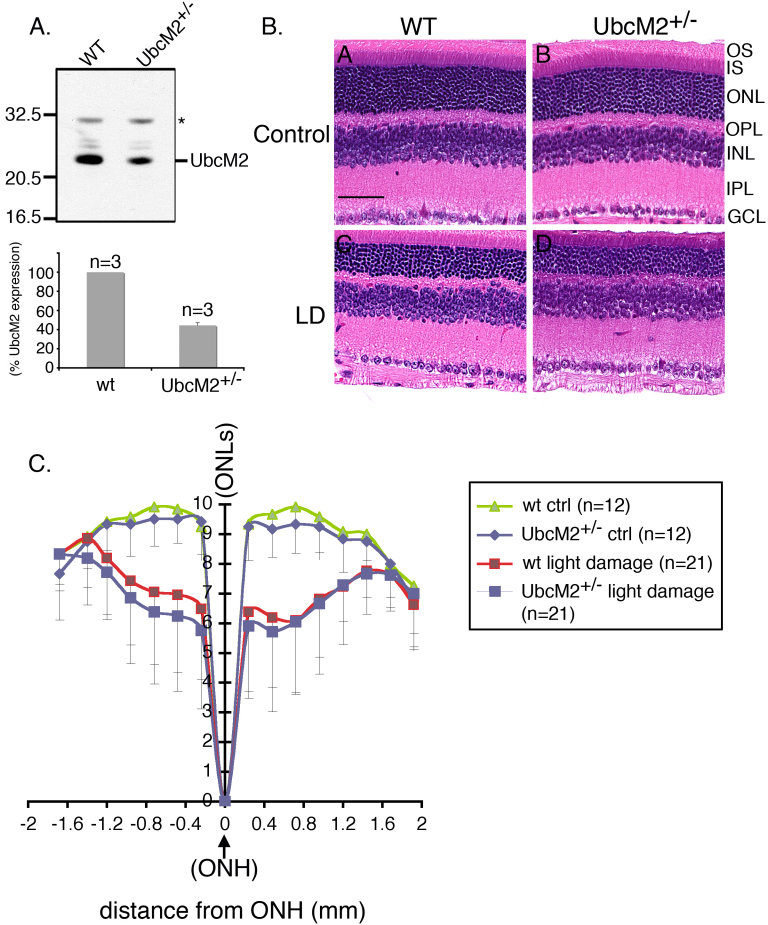
Mice harboring a single intact allele of UbcM2 are not more susceptible to light-induced retinal degeneration. **A**: Inactivation of a single UbcM2 allele reduces expression of the enzyme by 58%. Equal amounts (10 μg) of retinal lysates derived from a UbcM2**^+/−^** mouse and a wild-type (WT) littermate were subjected to denaturing and reducing sodium dodecyl sulfate PAGE (SDS–PAGE) followed by anti-UbcM2 western blotting. The migration of molecular weight markers is shown on the left. The asterisk denotes a nonspecific band serving as a loading control. The graph depicts the relative level of expression of UbcM2 in WT versus heterozygous littermates (n=3 of each genotype) as determined using a desktop scanner and Image J software. **B**: Representative hematoxylin and eosin (H&E) stained paraffin-embedded sections are shown from mice (UbcM2**^+/−^** and WT littermates) 7 days after acute bright-light challenge. Control denotes animals that were maintained in dim light for the entire experiment. Abbreviations of retinal cell layers are as described in the legend for Figure 3. The magnification bar in panel A represents 50 μm. **C**: A Spider graph representing data compiled from the indicated number of animals for each experimental condition. The error bars represent the standard deviation. Outer nuclear layer (ONL) rows are plotted along the *y*-axis with inferior and superior distances in mm from the optic nerve head (ONH) depicted along the *x*-axis. There was no statistically significant difference in ONLs between WT and UbcM2**^+/−^** control animals or between WT and UbcM2**^+/−^** light-damaged animals.

To test for a potential antioxidant/protective function of UbcM2 in the retina, we used an established bright-light damage model of acute oxidative stress and retinal degeneration [37,40,41]. In this assay, albino mice are reared in the dark and then exposed to 3,000 lux of cool white light for 6 h. Exposure to the light induces an oxidative stress that is lethal to photoreceptors. Thus, by counting the rows of nuclei in the ONL a week after light exposure (to allow clearance of the dead photoreceptors), the susceptibility of the retina to photo-oxidative insult can be quantified and evaluated. To determine if a full complement of UbcM2 is required for countering oxidative stress in the retina, we compared the toxicity of the light challenge in mice heterozygous for UbcM2 versus wild-type littermates. Disruption of a single UbcM2 allele reduces expression of the enzyme in the retina by 58±3% (Figure 4A, graph). We were unable to perform these experiments in UbcM2 null mice as disruption of both alleles is embryonic lethal (unpublished results). Control animals were maintained in dim light for the entire experiment. Isolated retinas were paraffin embedded, sectioned, stained with hematoxylin and eosin (Figure 4B), and then analyzed for the number of layers comprising the ONL (Figure 4C). From these experiments, we found that 3,000 lux was sufficient to induce the loss of three to four layers of nuclei flanking the ONH in both UbcM2 heterozygotes and their wild-type littermates (Figure 4B-D). Interestingly, we observed that UbcM2 heterozygote females displayed an apparently higher sensitivity to the bright-light challenge, although this difference was not statistically significant (data not shown). These data reveal that a 58% reduction in UbcM2 levels does not increase the susceptibility of the retina to acute photo-oxidative toxicity, as measured by bright-light-induced photoreceptor death.

**Figure 5:**
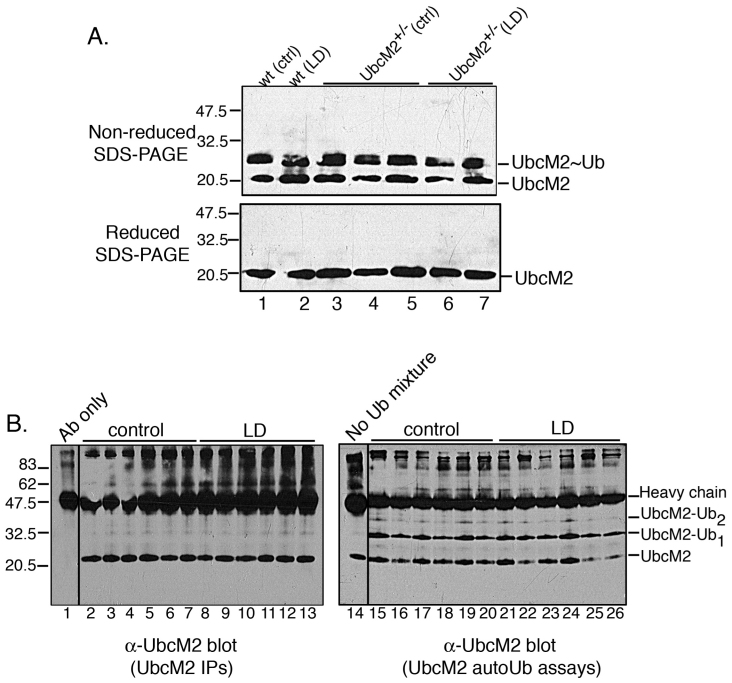
Acute bright-light stress does not reduce the steady-state level of Ub-charged UbcM2 or the catalytic activity of the enzyme. **A**: Lysates derived from the retinas of wt mice (lanes 1 and 2) and UbcM2**^+/−^** mice (lanes 3–7) maintained in dim light (lanes 1, 3, 4, 5) or exposed to 3,000 lux for 6 h (lanes 2, 6, 7) were resolved by nonreducing (top blot) or reducing (bottom blot) sodium dodecyl sulfate PAGE (SDS–PAGE) followed by anti-UbcM2 western blotting. Ub-charged enzyme is evident as a slower migrating band in nonreducing SDS–PAGE (top blot, indicated by “UbcM2~Ub”). This band collapses to uncharged UbcM2 in samples exposed to reducing agent (bottom blot). Each lane represents lysate from an individual mouse. **B**: Left blot— Shown in this blot is the enzyme used in the auto-ubiquitylation assay. The enzyme was immunoprecipitated from UbcM2+/− retinal lysates. Each lane corresponds to lysate from an individual mouse. Lane 1 contains antibody (Ab) only to distinguish bands derived from the Ab versus those IPed by the Ab. Right blot—IPed UbcM2 was combined with recombinant E1, Ub, and energy and incubated at 37 °C for 90 min. A control lacking the auto-ubiquitylation reaction mixture is shown in lane 14. Reaction products were analyzed by SDS–PAGE and anti-UbcM2 western blotting. UbcM2-Ub_1_ denotes a band that corresponds to mono-ubiquitylated UbcM2, and UbcM2-Ub_2_ denotes a band that corresponds to two Ub molecules conjugated to the enzyme. Vertical lines between lanes 1 and 2 (left blot) and lanes 14 and 15 (right blot) indicate lanes were not adjacent on original blots. Molecular weight markers were run between samples 1 and 2 and samples 14 and 15. The migration of heavy chain is indicated, and the migration of molecular weight markers is shown on the left for (**A**) and (**B**).

We also analyzed the catalytic state of UbcM2 in response to bright-light stress. The catalytic state of an E2 is reflected by the relative amount of enzyme that is charged with Ub on its active site cysteine. For these experiments, mice were either maintained in dim light or exposed to 3,000 lux for 6 h and sacrificed. Retinal lysates were generated under nonreducing conditions to maintain the thiolester linkage between the active site cysteine of UbcM2 and the COOH-terminal glycine of Ub. The lysates were subsequently split in half and resolved in parallel by nonreducing and reducing SDS–PAGE followed by anti-UbcM2 western blotting. The rationale for this methodology is that the attachment of Ub to the active site cysteine results in a slower migrating, ~30-kDa band in nonreducing SDS–PAGE. The addition of β-mercaptoethanol reduces the thiolester bond, thereby removing Ub from the active site and collapsing the 30-kDa band to the faster migrating 23-kDa band (Figure 5A, compare top and bottom blots) [19]. These experiments revealed that in both WT and UbcM2^+/−^ retinas, the level of Ub-charged UbcM2 was maintained in the bright-light challenged retinas as compared to the dim-light control samples (Figure 5A, compare lanes 2,6,7 to lanes 1,3,4,5). This result is somewhat surprising considering that previous studies show particular E2s lose Ub from their active sites in response to oxidative stress (e.g., [39]).

We next performed auto-ubiquitylation assays using UbcM2^+/−^ retinal lysates to determine if the capacity of the enzyme to transfer Ub was affected by an acute oxidative stress. Of note, heterozygote mice were chosen for these experiments because the data in Figure 4 implied that expression of the enzyme from a single intact allele provided sufficient catalytic activity to protect photoreceptors from an acute photo-oxidative stress. For this assay, UbcM2 was immunoprecipitated from retinal lysates derived from six control animals and six light-challenged animals (Figure 5B, left panel) and combined with recombinant E1, Ub, and an ATP-regenerating system for 90 min at 37 °C. Reaction products were resolved by SDS–PAGE and analyzed by anti-UbcM2 western blotting. Auto-ubiquitylation of UbcM2 is detected by the generation of a slower migrating band(s) in this assay. These experiments showed that UbcM2 auto-ubiquitylation was comparable between the control and light-challenged samples (Figure 5B, lanes 15–20 versus 21–26). Notably, by far the most prominent product of the auto-ubiquitylation reaction was mono-ubiquitylated UbcM2, in agreement with previously reported results for this enzyme [42]. These data show that UbcM2 retains an intact active site cysteine (i.e., not oxidized) following an acute photo-oxidative insult and can therefore function in the in vitro assay to get charged with Ub and undergo auto-ubiquitylation. Identical results were obtained with UbcM2 isolated from wt retinas (not shown). Together, the thiolester and ubiquitylation data reveal that in response to acute bright-light challenge, the steady-state levels of Ub-charged UbcM2 remain constant and the enzyme retains its capacity to transfer Ub, indicating maintenance of an intact and functional active site.

## Discussion

In this report we describe the expression and distribution of the class III Ub-conjugating enzymes in the retina. These three enzymes share 95% identity in their 150 amino acid catalytic core domains and are thus principally distinguished at the amino acid level by their unique N-terminal domains [17]. We have determined that these enzymes are further distinguished by their apparent cellular distribution in the retina. Specifically, UbcM2 appears to be the most highly expressed of the three, followed by UbcM3 and then UBE2E2 (Figure 1B and Figure 3C,F,I). These expression patterns and levels appear to be established in young mice and sustained as the mice age (Figure 3A-I). These conclusions are derived from data generated with rigorously characterized affinity-purified antibodies specific for each enzyme (Figure 1A). Of the three enzymes, UbcM2 is also the only one we detected in the nuclei of RPE cells and in photoreceptors (Figure 3B,C,F,I). This relative enrichment of UbcM2 in the retina was further demonstrated by an analysis of the enzyme’s distribution in different mouse tissues and organs (Figure 2).

Despite the discovery of numerous E3 ligases that can interact with UbcM2 [20,21,43], to date no substrates have been identified; thus the function(s) of the enzyme remains an open question. Previous work in the yeast *S. cerevisiae* established that a pair of nearly identical E2s, referred to as Ubc4/5, mediates the degradation of oxidatively damaged and misfolded proteins [17,22-24]. Furthermore, exogenous UbcM2 expression partially rescued the cold-sensitive growth phenotype of a yeast strain lacking Ubc4/5 [17]. An implication of these findings is that UbcM2 functions in mammalian cells to ubiquitylate oxidatively damaged and misfolded proteins and thereby targets these potentially toxic species for destruction by the 26S proteasome. We attempted to test this idea using a bright-light stress model of photo-oxidative stress [37,40,41]. These studies were done by comparing the toxicity of acute bright light to photoreceptors in mice harboring a single intact UbcM2 allele versus their wild-type littermates. We were unable to generate homozygous UbcM2-knockout mice for these studies because complete loss of the enzyme is embryonic lethal (unpublished data). Although inconvenient with respect to studying the requirement for the enzyme in retinal function, this lethality phenotype reveals that the class III E2s (UbcM2, UBE2E2, and UbcM3) have one or more nonredundant functions during development. Disruption of a single UbcM2 allele reduced expression of the enzyme by 58% in the retina (Figure 4A), but this did not result in a statistically significant increase in the susceptibility of photoreceptors to bright-light-induced death (Figure 4B,C). We interpret these data to indicate that either the remaining UbcM2 is sufficient to handle the oxidative burden imposed on the photoreceptors in this model of photo-oxidative stress (consistent with the data in Figure 5), and/or other E2s, such as the UbcH5 family (UbcH5a, UbcH5b, UbcH5c), compensate for the reduced levels of UbcM2 in the heterozygous animals. The UbcH5 family of E2s is an attractive candidate as these enzymes are also homologous to yeast Ubc4/5 [44] and have been linked to the turnover of damaged proteins (e.g., [23,43,45,46]). Based on the relatively high oxidative stress environment of the retina [1], it is reasonable to assume that multiple enzymatic components of the UPS contribute to the turnover of damaged proteins.

Our finding that acute bright-light challenge did not markedly alter the level of Ub-charged UbcM2 (i.e., activated enzyme; Figure 5A) may indicate that loaded enzyme is in a complex that shields it from inactivation by reactive oxygen species. Consistent with this notion is our previous observation that Ub-charged UbcM2 is selectively bound by the nuclear transport receptor, importin-11 [19]. Strikingly, acute oxidative stress also did not detectably influence the capacity of UbcM2 to be subsequently charged with and transfer Ub in vitro (Figure 5B). Although oxidative stress can modify, and often inactivate, surface-exposed cysteine residues of enzymes (e.g., [39,47-49]), our data show that the active site of UbcM2 is largely protected from irreversible oxidative inactivation by bright-light stress. This could be due to sheltering of the active site by a binding partner or disulfide bonding. This property of UbcM2 to retain a functional active site cysteine in the presence of bright-light stress may explain the robust expression of the enzyme in the retina as it would enable the enzyme to retain its capacity to protect the retina from the toxic accumulation of oxidatively damaged proteins in the face of chronic exposure to bright light. This retention of catalytic activity may also explain why inactivation of a single UbcM2 allele was not sufficient to increase the susceptibility of the photoreceptors to the photo-oxidative insult (Figure 4B,C).

In summary, we demonstrate for the first time the expression patterns of the class III E2s in the retina using specific antibodies for each enzyme. We show that among these enzymes, UbcM2 is the most robustly expressed and appears to be further distinguished by its expression in photoreceptors and RPE cells. Inactivation of a single UbcM2 allele did not increase the sensitivity of photoreceptors to acute oxidative stress-induced lethality, which may reflect the existence of compensating E2s and/or that the remaining UbcM2 activity was sufficient to tag oxidatively damaged proteins for destruction. Our data also decisively show that although UbcM2 is ubiquitously expressed, it is most prominently expressed in the retina and thus warrants further analysis. These data represent an essential step in understanding and dissecting the contributions of E2s in countering the chronic oxidative burden that threatens the health of vulnerable cells of the retina.
